# Transcranial direct current stimulation combined with cognitive training for working memory in healthy adults: a systematic review and robust variance meta-analysis of trained and untrained outcomes

**DOI:** 10.3389/fnhum.2026.1902938

**Published:** 2026-08-03

**Authors:** Hong-Qi Wang, Yan Jiang, Ling-Ling Zhu, Ming Fan, Yong-Qi Zhao, Du-Ming Wang

**Affiliations:** 1 Department of Psychology, Zhejiang Sci-Tech University, Hangzhou, China; 2 Beijing Institute of Basic Medical Sciences, Beijing, China; 3 Information Sciences & Engineering, Lanzhou University, Lanzhou, China

**Keywords:** cognitive training, healthy adults, meta-analysis, robust variance estimation, systematic review, tDCS, transcranial direct current stimulation, working memory

## Abstract

**Introduction:**

Transcranial direct current stimulation (tDCS) has been proposed as a potential tool for enhancing the benefits of cognitive training, but its added value for working memory in healthy adults remains unclear. This systematic review and robust variance estimation meta-analysis examined whether active tDCS combined with cognitive training improves post-training working memory performance compared with sham tDCS combined with the same training, and whether the effect varies by outcome type, assessment time point, and training characteristics.

**Methods:**

PubMed, Scopus, and Web of Science were searched from database inception to January 2026. Eligible studies were randomized controlled trials involving healthy adults, comparing active tDCS plus structured cognitive training with sham tDCS plus otherwise identical training, and reporting at least one post-training working memory outcome. Multiple correlated effect sizes within studies were synthesized using robust variance estimation. Outcomes were classified as trained-task outcomes or untrained working-memory outcomes, and both immediate post-test and follow-up assessments were included.

**Results:**

A total of 23 studies contributing 135 effect sizes were included. Active tDCS combined with cognitive training produced a small but statistically significant improvement in post-training working memory performance, Hedges’ *g* = 0.191, 95% CI [0.062, 0.320], *p* = 0.006. Effects were significant for trained-task outcomes, whereas effects on untrained working-memory outcomes were smaller and not statistically significant; however, the direct difference between the two outcome categories was not significant. Immediate post-test effects were significant, whereas follow-up effects remained positive and similar in magnitude but lay at the conventional alpha threshold, with no significant difference between time points. Stimulation timing, age group, and training type did not significantly moderate the effect. Sensitivity analyses confirmed the stability of the overall estimate, and no clear evidence of small-study effects was detected.

**Discussion:**

These findings suggest that tDCS may provide a small adjunctive benefit when combined with cognitive training in healthy adults, with the most consistent benefits observed for trained-task performance and more limited evidence for transfer to untrained working-memory outcomes.

## 1 Introduction

Working memory refers to the capacity to maintain, update, and manipulate information over short periods of time, and it provides an essential foundation for higher-order cognitive processes such as reasoning, learning, comprehension, and problem solving (Baddeley, 2012). From the perspective of cognitive neuroscience, working memory is not a unitary construct but is supported by a multicomponent system and its underlying distributed neural networks. Within this network, the prefrontal cortex plays a central role in maintaining goal representations, regulating attentional resources, and coordinating task-relevant information (D'Esposito and Postle, 2015; Smith and Jonides, 1997; Wager and Smith, 2003). For this reason, working memory has long been regarded as a key cognitive domain in studies of cognitive training and neuromodulation.

Given the central role of working memory in a wide range of cognitive activities, researchers have extensively attempted to improve working memory performance through cognitive training, with the further expectation that training-related gains may transfer to untrained tasks or broader cognitive domains. However, existing evidence suggests that the benefits of cognitive training do not consistently translate into broad transfer effects. This issue is particularly evident in healthy adults, for whom improvements in the trained task are often stronger than improvements in untrained tasks (Melby-Lervåg and Hulme, 2013; Soveri et al., 2017; Shipstead et al., 2012). In healthy older adults, working memory training may produce a certain degree of near transfer and even delayed benefits, but the overall effects are typically small to moderate and vary substantially across tasks, populations, and training protocols (Melby-Lervåg and Hulme, 2013; Soveri et al., 2017). Therefore, how to improve the efficiency of cognitive training and enhance transfer to untrained working-memory tasks remains an important question in this field.

Transcranial direct current stimulation (tDCS) is a non-invasive brain stimulation technique that modulates cortical excitability and neural plasticity by applying a weak direct current through electrodes placed on the scalp. Seminal work has shown that weak direct current stimulation can alter the excitability state of cortical neurons (Nitsche and Paulus, 2000), and subsequent reviews have further suggested that tDCS may modulate learning and cognitive performance by influencing cortical functional states, synaptic plasticity, and task-related network activity (Filmer et al., 2014). Because working memory depends strongly on prefrontal networks, particularly the dorsolateral prefrontal cortex (DLPFC), which plays an important role in the maintenance, manipulation, and control of working-memory representations, the prefrontal cortex—especially the left or right DLPFC—has become one of the most common stimulation targets in tDCS studies of working memory (Barbey et al., 2013; D'Esposito and Postle, 2015). In healthy populations, however, findings regarding the effects of tDCS alone on working memory remain controversial. Early studies and reviews suggested that tDCS may facilitate working memory (Brunoni and Vanderhasselt, 2014; Fregni et al., 2005; Hill et al., 2016), but conclusions across meta-analyses have not been fully consistent, indicating that single-session stimulation, task characteristics, stimulation parameters, and sample heterogeneity may substantially influence the observed effects (Horvath et al., 2015; Mancuso et al., 2016). To address the limited and unstable effects of tDCS when applied alone, and to explore its potential synergy with activity-dependent plasticity, an increasing number of studies have combined tDCS with cognitive training. The underlying rationale is that if tDCS can enhance task-related neural activity or increase neural plasticity during training, its effect may not primarily manifest as an isolated, one-time cognitive enhancement, but rather as an amplification of training-related gains.

Empirical studies have shown that tDCS combined with working memory training or other forms of cognitive training can, under certain conditions, improve the learning curve of the trained task and may promote transfer to untrained tasks. For example, Ruf et al. found in healthy adults that DLPFC tDCS enhanced the benefits of working memory training, and that these benefits may transfer to untrained tasks or cognitive abilities (Ruf et al., 2017). Ke et al. (2019) also reported in a healthy young adult sample that tDCS may influence performance changes across repeated sessions of working memory training. At the same time, other studies have shown that such facilitative effects are not stable, and that their direction and magnitude may be influenced by factors such as training type, stimulation target, stimulation timing, age group, and outcome measure (Au et al., 2016; Martin et al., 2014; Richmond et al., 2014; Trumbo et al., 2016). Consequently, individual studies are often insufficient to support generalizable conclusions.

In recent years, preliminary synthetic evidence has emerged regarding whether tDCS combined with cognitive training provides benefits beyond cognitive training alone. Pergher et al. conducted a systematic review and meta-analysis of concurrent tDCS and working memory training, reporting that tDCS may yield additional benefits for both trained and untrained tasks under certain conditions, with particular potential for enhancing transfer effects (Pergher et al., 2022). In addition, a meta-analysis by Lv et al. (2024) focusing on healthy older adults suggested that tDCS combined with cognitive training may improve working memory performance relative to control conditions. However, several limitations remain in the existing evidence syntheses. First, some reviews have focused primarily on working memory training itself and have not incorporated broader forms of cognitive training. Second, some syntheses have been restricted to healthy older adults, limiting the generalizability of their conclusions to the broader healthy adult population. Third, many reviews have not clearly distinguished between trained-task outcomes and untrained working-memory outcomes, making it difficult to determine whether tDCS is more likely to enhance training-specific gains or transfer-related benefits. Fourth, conventional meta-analyses often retain only one effect size per study, which makes it difficult to adequately address dependency arising from multiple tasks, outcome measures, and assessment time points within the same study.

Against this background, a more refined synthesis is needed to evaluate the effects of active tDCS combined with cognitive training compared with sham tDCS combined with the same cognitive training in healthy adults, both in terms of the research question and the statistical approach. The present study therefore aimed to systematically examine whether active tDCS combined with cognitive training produces additional improvements in post-training working memory performance in healthy adults relative to sham tDCS combined with the same training, and whether this effect varies as a function of outcome type, assessment time point, and other study characteristics.

Compared with previous reviews, the present study makes three main contributions. First, whereas existing reviews have largely focused on tDCS combined with working memory training, this study extends the inclusion scope to broader forms of cognitive training, thereby allowing a more comprehensive evaluation of the boundary conditions under which tDCS may serve as a training-enhancement tool. Second, this study distinguishes post-training working memory outcomes into trained-task outcomes and untrained working-memory outcomes, enabling separate examination of task-specific gains and transfer-related benefits. This distinction allows for a more direct assessment of whether tDCS is more likely to amplify gains in the trained task itself or to enhance broader transfer to working memory performance. Finally, because primary studies often report multiple correlated effect sizes, this study uses robust variance estimation meta-analysis to account for within-study dependency. This approach allows more information from the original studies to be retained while reducing potential bias introduced by the subjective selection of a single effect size from each study (Hedges et al., 2010; Pustejovsky and Tipton, 2022; Tipton, 2015).

Thus, the present study is not intended as a simple replication of previous reviews. Rather, it seeks to re-evaluate the effects of tDCS combined with cognitive training on post-training working memory performance in healthy adults within a broader training scope, with a more refined classification of outcome domains, and under a statistical framework better suited to dependent multiple-outcome structures. In doing so, this study aims to provide clearer evidence regarding the boundaries of this intervention effect.

## 2 Methods

This systematic review and meta-analysis was conducted and reported in accordance with the Preferred Reporting Items for Systematic Reviews and Meta-Analyses (PRISMA) 2020 statement and the PRISMA-S extension for literature searches (Page et al., 2021; Rethlefsen et al., 2021). The review was not prospectively registered. To improve transparency, the manuscript and Supplementary material provide the eligibility criteria, full database search strategies, outcome-classification rules, effect-size dataset, risk-of-bias judgments, and complete R code.

### 2.1 Search strategy

Systematic searches were conducted in PubMed, Scopus, and Web of Science from database inception to January 2026. Because studies combining tDCS with cognitive training vary substantially in how cognitive outcomes are described, and because some eligible studies report working-memory outcomes without explicitly using the term “working memory” in the title, abstract, or keywords, a broad-search and refined-screening strategy was adopted.

The following search string was applied in each database: (“transcranial direct current stimulation” OR “tDCS”) AND (“practice” OR “training” OR “cognitive intervention”). No additional working-memory-related terms were added at the initial search stage, in order to reduce the risk of excluding studies that reported working-memory outcomes but did not explicitly identify them as such in the title, abstract, or keywords. The exact search string, database searched, search date, and number of records retrieved from each database are provided in Supplementary file 1.

**Figure 1:**
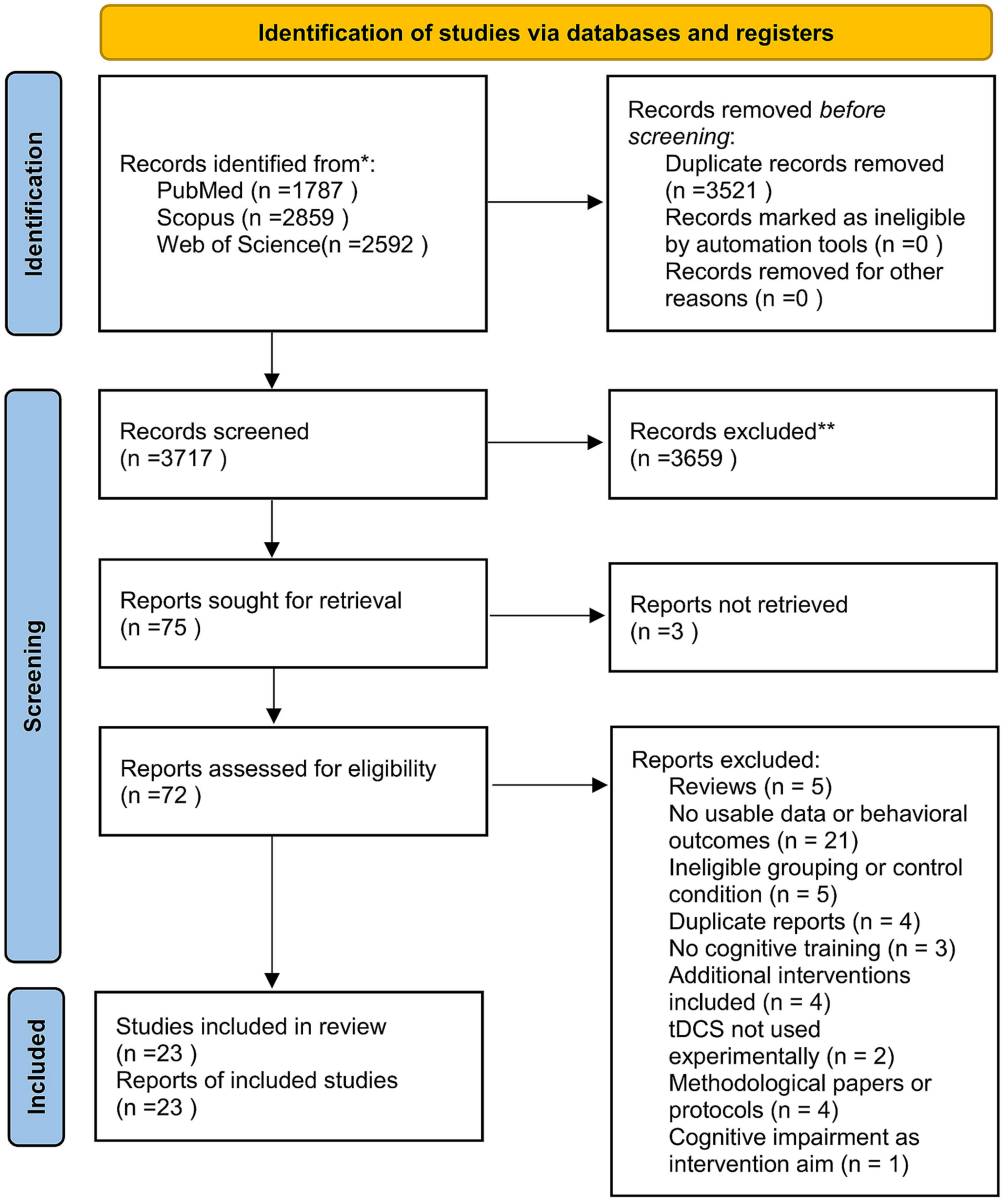
PRISMA 2020 flow diagram showing the study identification, screening, eligibility assessment, and inclusion process.

To reduce the risk of missing relevant studies, the reference lists of included studies and relevant reviews were also screened manually. All retrieved records were imported into reference management software, and duplicates were removed before title/abstract and full-text screening. Reasons for exclusion were recorded during full-text screening. The study selection process is presented in the PRISMA 2020 flow diagram (Figure 1). PsycINFO, Embase, CENTRAL, trial registries, dissertation databases, preprint servers, and other gray-literature sources were not searched because of practical constraints in database access and review resources.

### 2.2 Eligibility criteria

Eligibility criteria were defined according to the PICOS framework. The population was healthy adults aged 18 years or older. The intervention was active transcranial direct current stimulation (tDCS) combined with structured cognitive training. Eligible cognitive training protocols included working memory training, non-working-memory cognitive training, and multicomponent cognitive training. The comparator was sham tDCS combined with the same or otherwise equivalent cognitive training protocol. Eligible outcomes included at least one post-training working memory outcome, including trained-task outcomes and/or untrained working-memory outcomes. Follow-up outcomes were included when available. Eligible studies were randomized controlled trials.

Outcome classification was based on direct exposure to the assessment task during training. A trained-task outcome was performance on the same task, task version, or explicitly practiced outcome used during the intervention. An untrained working-memory outcome was any eligible working-memory measure that was not directly practiced during training. This exposure-based distinction was not intended to be equivalent to a near-transfer versus far-transfer taxonomy. The untrained category included both highly similar measures (for example, an untrained visuospatial n-back after verbal n-back training) and more distinct working-memory measures such as digit span, operation span, block-tapping, letter updating, or change-detection tasks. Because primary reports did not use a consistent transfer-distance taxonomy and the number of studies was limited, near and far transfer were not analyzed separately.

Studies were excluded if they involved clinical patient samples, children or adolescents, animal experiments, mixed samples from which healthy adult data could not be separated, studies without a sham tDCS control or comparable control condition, single-group pre-post designs, reviews, theoretical articles, editorials, or conference abstracts and other reports from which the required outcome data could not be obtained. When multiple reports referred to the same study, they were integrated and treated as a single study to avoid duplicate inclusion. Studies were not included in the main quantitative synthesis if the direction of the outcome could not be determined, if key statistical information was severely incomplete and could not be supplemented, or if there was a clear risk of selective outcome reporting.

### 2.3 Study selection, data extraction, and risk-of-bias assessment

One reviewer initially conducted the complete title/abstract screening, full-text eligibility assessment, data extraction, and risk-of-bias assessment. Uncertainties were discussed with the supervisory authors until consensus was reached, and the final list of included studies, extracted effect-size data, and risk-of-bias judgments were checked by the author team before analysis.

To assess the reliability of these procedures, a second reviewer independently reassessed random samples of 929 title/abstract records, 18 full-text reports, six included studies for data extraction, and 10 study-outcome entries for RoB 2 assessment. Agreement was 96.8% for title/abstract screening (899/929; Cohen’s *κ* = 0.848), 94.4% for full-text eligibility assessment (17/18; *κ* = 0.880), 98.5% for data extraction (745/756 fields), and 98.0% across the five RoB 2 domains (49/50 judgments; *κ* = 0.961). Disagreements were resolved through discussion and re-examination of the original reports.

Data extraction covered five domains. First, basic study information was extracted, including first author, publication year, country or region, and study design. Second, sample characteristics were extracted, including sample sizes of the active and sham groups, mean age and standard deviation, and age-group classification. Third, intervention characteristics were extracted, including training type, training task, whether the training was adaptive, number of training sessions, timing of tDCS, anode location, cathode location, stimulation target, current intensity, stimulation duration, electrode size, and blinding information. Fourth, outcome information was extracted, including trained-task outcomes, untrained working-memory outcomes, follow-up outcomes, outcome type, assessment time point, and statistical information required for effect-size calculation. Fifth, information relevant to risk of bias was extracted, including randomization, deviations from intended interventions, missing outcome data, outcome measurement, and selective reporting.

When the required data were not fully reported in the original article, study authors were contacted where possible. If the data remained unavailable, effect sizes were obtained where possible by using WebPlotDigitizer or by converting statistics such as *F* values from analysis of variance into effect sizes (Lakens, 2013; Rohatgi, 2017).

Risk of bias was assessed using the RoB 2 tool (Sterne et al., 2019). Risk of bias was evaluated across five domains: the randomization process, deviations from intended interventions, missing outcome data, measurement of the outcome, and selection of the reported result. Each study-outcome entry was rated as low risk of bias, some concerns, or high risk of bias, and an overall judgment was derived using the RoB 2 algorithms. Under RoB 2, “some concerns” is not a numerical label for moderate risk; it indicates that at least one domain raises some concern while no domain is judged high risk. An overall high-risk judgment is assigned when at least one domain is high risk or when multiple domain-level concerns substantially lower confidence in the result. The overall risk-of-bias level was further examined in sensitivity analyses to determine whether entries rated high risk systematically influenced the overall estimate.

### 2.4 Effect size calculation and statistical analysis

All statistical analyses were conducted in the R environment, primarily using the metafor and clubSandwich packages, with additional auxiliary packages used for data management and visualization when necessary (Pustejovsky and Tipton, 2022; Viechtbauer, 2010). For continuous outcomes, when different studies used different working memory measures that reflected the same underlying construct, the standardized mean difference (SMD) was used as the primary effect size, with Hedges’ g applied for small-sample correction. The direction of all effect sizes was coded consistently, such that positive values indicated better performance in the active tDCS combined with cognitive training condition than in the sham tDCS combined with the same cognitive training condition. For outcomes such as reaction time, where lower values indicate better performance, scores were reverse-coded before effect size calculation. When multiple estimates were available, we prioritized post-intervention between-group means and standard deviations. Change scores were used only when post-intervention data were unavailable, and adjusted estimates only when no other between-group data could be extracted.

Because individual studies often included multiple tasks, outcome measures, and assessment time points, the resulting effect sizes were not statistically independent. Therefore, a random-effects robust variance estimation (RVE) meta-analytic framework was used as the primary analytic approach (Hedges et al., 2010; Tipton, 2015; Tipton and Pustejovsky, 2015). Specifically, dependency among effect sizes was modeled by treating study as the clustering unit. RVE was then used to obtain cluster-robust standard errors and statistical inferences without mechanically averaging multiple outcomes within each study in advance. Shared-control comparisons, crossover contrasts, and repeated follow-up assessments were retained as separate effect sizes and treated as dependent within study clusters. For change-score effects, baseline–post-intervention dependence was preserved by using the reported change-score statistics.

For model implementation, a variance–covariance matrix was first constructed based on the sampling variance of each effect size, with the within-study correlation set to *ρ* = 0.50 in the primary analysis. Additional sensitivity analyses were then conducted with *ρ* = 0.10 and *ρ* = 0.90 to examine the robustness of the results to different assumptions about within-study correlation. Multivariate random-effects models were fitted using rma.mv, and robust testing procedures were applied to obtain robust standard errors, 95% confidence intervals, and significance tests for the overall effect and moderator effects (Hedges et al., 2010; Tipton, 2015; Viechtbauer, 2010). Robust tests used the CR2 adjustment with Satterthwaite small-sample degrees of freedom, and heterogeneity was estimated using REML variance components at the study and effect-size levels.

The primary model first fitted an intercept-only random-effects RVE model to estimate the overall effect on post-training working memory performance. Moderator analyses were then conducted within the same framework by adding covariates to the model. Based on the research questions and theoretical relevance, three primary moderators were examined. The first was outcome type, namely trained-task outcomes versus untrained working-memory outcomes. The second was assessment time point, namely immediate post-test versus follow-up. The third was training type, namely working memory training versus non-working-memory or mixed training. In addition to these primary moderators, stimulation timing and age group were further examined as exploratory moderators. Variables with clearly insufficient numbers of studies were not subdivided excessively, to avoid model overfitting.

Robustness analyses included changing the assumed within-study correlation, repeating the analyses after excluding high-risk studies, and comparing whether the results were consistent across different effect size extraction strategies. Publication bias was assessed using a two-step approach. First, funnel plots were generated based on study-level aggregated effect sizes for visual inspection. Second, given the potential mechanical association between SMDs and their conventional standard errors, Egger-type tests were preferentially conducted using a modified precision index defined on the basis of sample size, rather than using the conventional standard error directly as the predictor (Pustejovsky and Rodgers, 2019).

## 3 Results

### 3.1 Characteristics of included studies

A total of 23 studies were included in the systematic review and meta-analysis. Fourteen primarily involved healthy young-adult samples and nine primarily involved healthy older-adult samples. Across studies with reported ages, active- and sham-group mean ages ranged from 18.75 to 36.10 years in the young-adult category and from 64.33 to 73.78 years in the older-adult category. All participants were healthy adults. Detailed characteristics are provided in Supplementary Table S1.

In terms of outcome composition, 9 studies reported trained-task outcomes, 19 studies reported untrained working-memory outcomes, and 10 studies further provided follow-up outcomes. These outcome domains were not mutually exclusive, because the same study could report trained-task outcomes, untrained working-memory outcomes, and follow-up outcomes simultaneously. Therefore, the sum of the number of studies across outcome categories exceeds the total number of included studies.

Most included studies used randomized parallel-group, sham-controlled designs and compared active tDCS combined with cognitive training against sham tDCS combined with the same or otherwise equivalent training. Eighteen of the 23 studies used online stimulation, three used mixed timing, and two used offline stimulation. Sixteen studies used working-memory-specific training, five used mixed or multidomain training, and two used non-working-memory training. Training exposure ranged from 1 to 20 sessions; stimulation intensity ranged from 1 to 2 mA and duration from 10 to 30 min. Most studies targeted prefrontal regions, particularly the left, right, or bilateral DLPFC. Overall, the evidence was heterogeneous in sample characteristics, stimulation parameters, training protocols, and outcome measures.

### 3.2 Risk of bias

**Figure 2:**
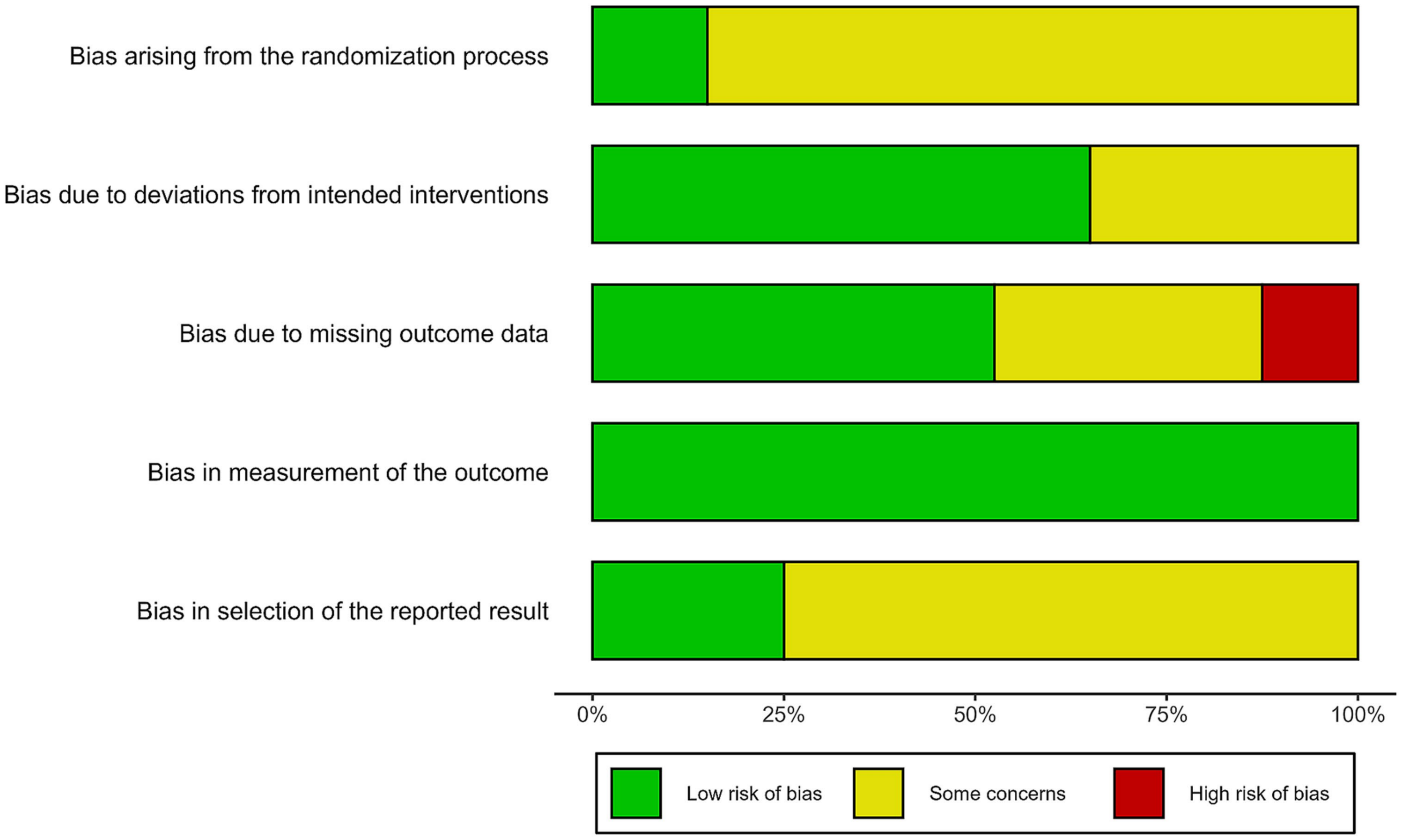
Risk-of-bias assessment based on the RoB 2 tool. Bars show the proportion of study-outcome entries rated as low risk of bias, some concerns, or high risk of bias across the five RoB 2 domains.

Risk of bias was assessed using the RoB 2 tool. Among the 40 study-outcome entries classified by outcome domain and assessment time point, 34 were rated as having some concerns, five as high risk of bias, and one as low risk of bias, as shown in Figure 2. Under the RoB 2 framework, a judgment of some concerns indicates that at least one domain raised a methodological concern, while the available information was not sufficient to support an overall high-risk judgment. Thus, although relatively few entries were rated as high risk, the limited number of low-risk entries indicates that methodological uncertainty remained across much of the evidence base.

A sensitivity analysis was conducted after excluding the five entries rated as high risk. The pooled effect remained statistically significant, Hedges’ g = 0.187, 95% CI [0.054, 0.319], *p* = 0.009, and was similar in magnitude to the primary estimate, Hedges’ *g* = 0.191, 95% CI [0.062, 0.320], *p* = 0.006. These findings suggest that the overall estimate was not substantially altered by the exclusion of high-risk entries, although the predominance of entries rated as some concerns should be considered when interpreting the strength of the evidence.

### 3.3 Overall effect

**Figure 3:**
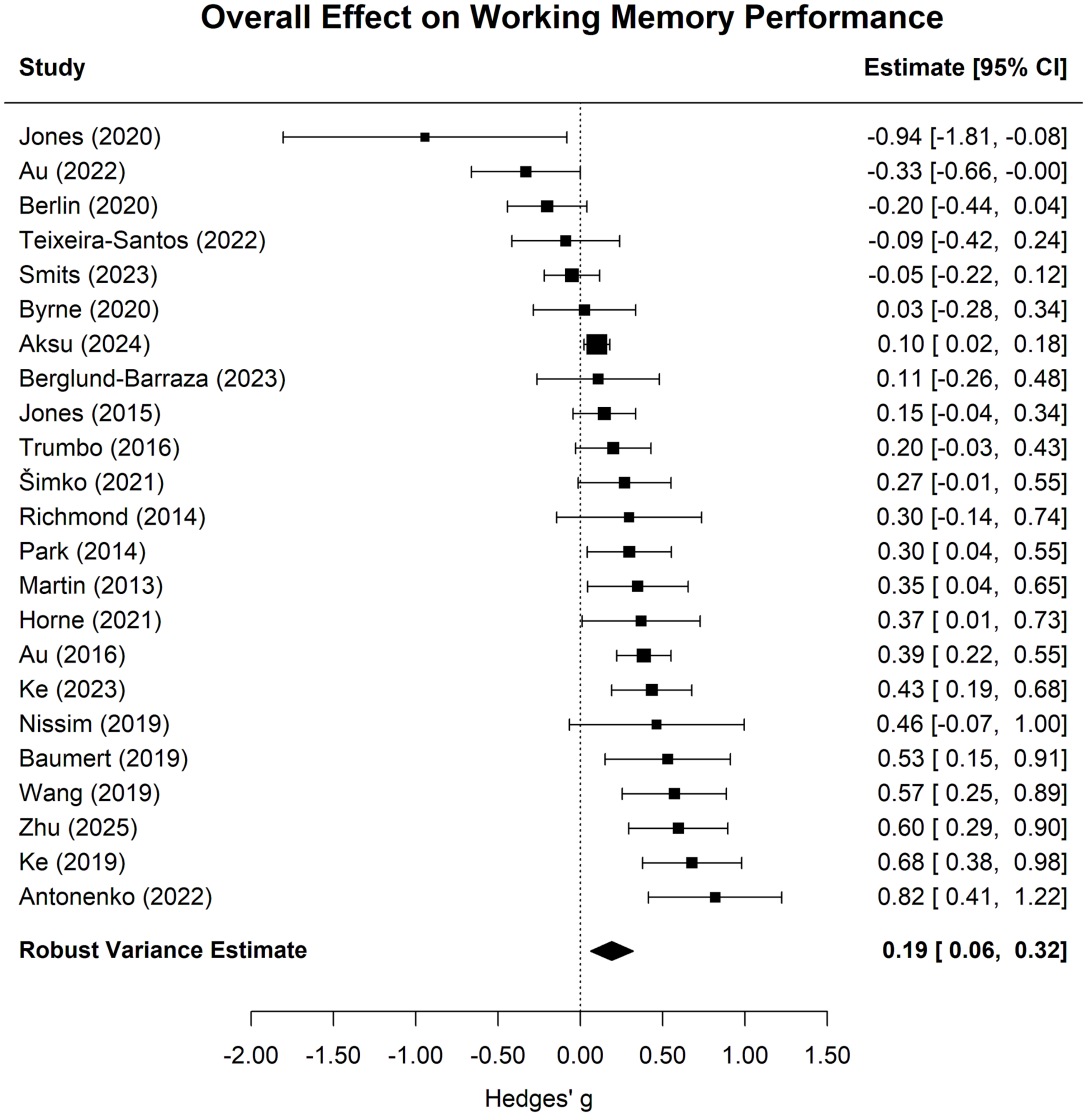
Overall effect of active transcranial direct current stimulation combined with cognitive training on post-training working memory performance in healthy adults. The primary robust variance estimation model included 135 effect sizes from 23 studies. Active tDCS combined with cognitive training produced a small but statistically significant additional benefit compared with sham tDCS combined with the same training, Hedges’ *g* = 0.191, 95% CI [0.062, 0.320], *p* = 0.006.

The overall RVE model showed that active tDCS combined with cognitive training significantly improved post-training working memory performance compared with sham tDCS combined with the same cognitive training, with an effect size of Hedges’ *g* = 0.191, SE = 0.061, 95% CI [0.062, 0.320], *df* = 15.82, *p* = 0.006. This result indicates that, at the overall level, tDCS produced a statistically significant but small additional working memory benefit beyond cognitive training alone, as shown in Figure 3.

The primary model included 23 independent study clusters and 135 effect sizes. The estimated variance components were *τ*^2^ < 0.001 at the study level and *τ*^2^ = 0.318 at the effect-size level, with significant residual heterogeneity, Q(134) = 572.37, *p* < 0.001. Residual variance components at the study/effect-size levels were <0.001/0.312 for outcome type, <0.001/0.322 for assessment time point, <0.001/0.325 for training type, <0.001/0.325 for stimulation timing, and <0.001/0.321 for age group. All primary and exploratory moderator models included 23 independent study clusters.

### 3.4 Primary moderator analyses

In the analysis by outcome type, 9 studies contributed 35 trained-task effect sizes and 19 studies contributed 100 untrained working-memory effect sizes; these categories were not mutually exclusive at the study level. The trained-task estimate was statistically significant, Hedges’ *g* = 0.424, SE = 0.099, 95% CI [0.180, 0.667], *p* = 0.005. The untrained working-memory estimate was positive but did not reach statistical significance, Hedges’ *g* = 0.130, SE = 0.078, 95% CI [−0.040, 0.301], *p* = 0.121. However, the direct contrast between the two outcome types was not statistically significant, *b* = −0.293, SE = 0.142, 95% CI [−0.626, 0.039], *df* = 7.34, *p* = 0.076. Thus, although the point estimate was larger for trained-task outcomes, the current evidence does not establish a reliable difference between outcome types.

In the analysis by assessment time point, the immediate post-test estimate was statistically significant, Hedges’ *g* = 0.195, SE = 0.067, 95% CI [0.053, 0.336], *p* = 0.010. The follow-up estimate was positive and lay at the conventional alpha threshold, Hedges’ *g* = 0.182, SE = 0.076, 95% CI [0.000, 0.364], *p* = 0.050. The difference between follow-up and immediate post-test estimates was not significant, *b* = −0.013, SE = 0.076, 95% CI [−0.189, 0.163], *df* = 8.05, *p* = 0.872. Thus, the estimates were similar in direction and magnitude across the two time points, although the follow-up estimate should be interpreted cautiously given its borderline statistical evidence and limited precision.

Given the small number of studies using non-working-memory training, training type was combined into two categories in the formal analysis to improve estimation stability: working memory training and non-working-memory or mixed training. The results showed that the pooled effect was statistically significant in the working memory training group, Hedges’ *g* = 0.182, SE = 0.077, 95% CI [0.015, 0.349], *p* = 0.035. The pooled effect in the non-working-memory or mixed training group was positive but did not reach statistical significance, Hedges’ g = 0.184, SE = 0.109, 95% CI [−0.181, 0.549], *p* = 0.198. Further meta-regression showed no significant difference between the non-working-memory or mixed training group and the working memory training group, *b* = 0.002, SE = 0.134, 95% CI [−0.333, 0.336], *df* = 5.51, *p* = 0.990. These results suggest that the current evidence does not support a significant moderating effect of training type on the intervention effect. Put differently, the additional effect of active tDCS did not appear to depend specifically on working memory training.

### 3.5 Exploratory moderator analyses

Because the number of studies using mixed and offline stimulation was limited, stimulation timing was further combined into two categories to improve model estimation stability: online stimulation and non-online stimulation. The results showed a statistically significant effect under online stimulation, Hedges’ *g* = 0.211, SE = 0.070, 95% CI [0.060, 0.362], *p* = 0.010. However, the difference between non-online and online stimulation was not significant, *b* = −0.096, SE = 0.152, 95% CI [−0.505, 0.312], *df* = 4.40, *p* = 0.559. Therefore, the current evidence does not indicate a significant moderating effect of stimulation timing on the intervention effect.

In the analysis by age group, the effect was statistically significant in young adults, Hedges’ *g* = 0.211, SE = 0.093, 95% CI [0.007, 0.416], *p* = 0.044. In older adults, the effect was positive but did not reach statistical significance, Hedges’ *g* = 0.166, SE = 0.073, 95% CI [−0.021, 0.353], *p* = 0.072. The difference between the two age groups was not significant, *b* = −0.046, SE = 0.118, 95% CI [−0.302, 0.210], *df* = 12.30, *p* = 0.705, suggesting that the current evidence does not support a significant moderating effect of age group on the intervention effect.

### 3.6 Sensitivity analyses

To examine the robustness of the model to assumptions about within-study correlation, the overall RVE analysis was repeated under three values of *ρ*: 0.10, 0.50, and 0.90. When *ρ* = 0.10, the overall effect was Hedges’ *g* = 0.222, 95% CI [0.099, 0.345], *p* = 0.001. When *ρ* = 0.50, the overall effect was Hedges’ *g* = 0.191, 95% CI [0.062, 0.320], *p* = 0.006. When *ρ* = 0.90, the overall effect was Hedges’ *g* = 0.178, 95% CI [0.038, 0.318], *p* = 0.016. Across different assumptions regarding within-study correlation, the direction of the effect remained consistent and all estimates were statistically significant. These findings indicate that the overall conclusion was not sensitive to the assumed within-study correlation and showed good robustness.

### 3.7 Publication bias

**Figure 4:**
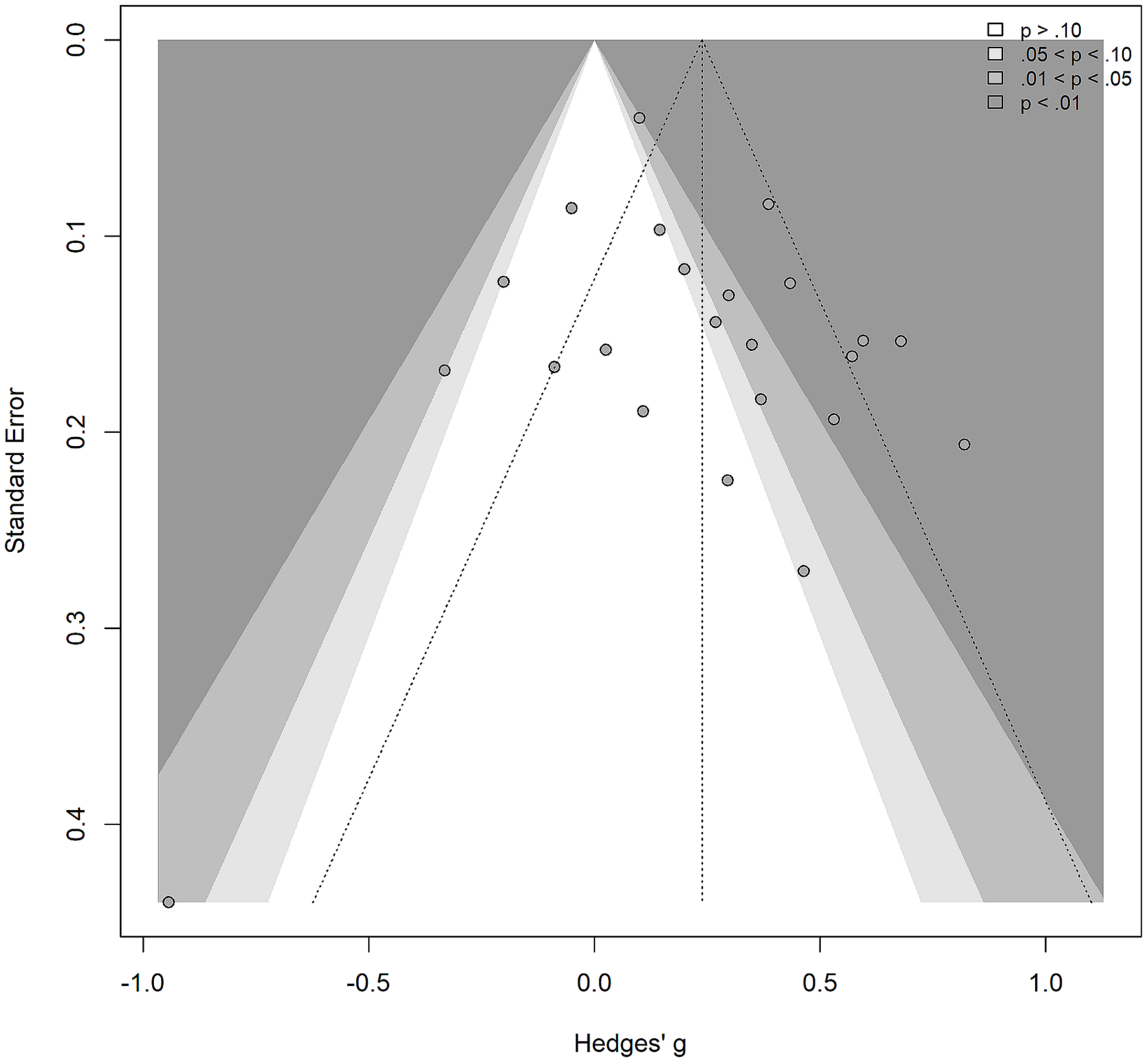
Funnel plot based on study-level aggregated effect sizes for visual inspection of potential publication bias or small-study effects.

To assess potential publication bias, funnel plots were first generated based on study-level aggregated effect sizes for visual inspection, as shown in Figure 4. Visual inspection did not reveal clear evidence of publication bias or other small-study effects. In addition, small-study effects were statistically examined using study-level Egger regression. The results showed that the slope of the precision index was not significant, *b* = −0.342, SE = 1.054, 95% CI [−2.408, 1.724], *p* = 0.746, suggesting no clear evidence of small-study effects. Given that the primary analysis used a multi-effect-size RVE framework and that standardized mean differences may be mechanically associated with conventional standard errors, a modified Egger regression under the RVE framework was further conducted using a sample-size-based modified precision index, sqrtW. The results showed that the slope of sqrtW was also not significant, *b* = 0.062, SE = 0.824, 95% CI [−2.717, 2.840], *p* = 0.945. Taken together, the available analyses did not provide clear evidence of small-study effects. However, given the limited number of included studies, publication bias cannot be ruled out.

## 4 Discussion

### 4.1 Principal findings and interpretation

Using a systematic review and robust variance estimation meta-analysis, the present study examined the effects of active tDCS combined with cognitive training on post-training working memory performance in healthy adults, compared with sham tDCS combined with the same training. The results showed that active stimulation produced a statistically significant but small additional benefit. The point estimate was larger for trained-task outcomes than for untrained working-memory outcomes, although the direct moderator contrast did not reach statistical significance and the trained-task estimate was based on a smaller number of studies. The follow-up estimate remained positive and was similar in direction and magnitude to the immediate post-test estimate, although its statistical evidence was less conclusive. No significant moderating effects were observed for training type, stimulation timing, or age group. Sensitivity analyses indicated that the overall estimate was stable across alternative analytic assumptions. Taken together, the findings suggest a small adjunctive benefit of tDCS when combined with cognitive training, while the interpretation of its boundary conditions should take into account the methodological characteristics and coverage of the available evidence.

Overall, the present findings suggest that tDCS may have a potentially useful adjunctive role in cognitive training rather than an independent and powerful cognitive enhancement intervention. This conclusion is consistent with recent cautious perspectives on non-invasive brain stimulation, which suggest that stimulation effects are more likely to depend on coupling with task processes, the individual’s current neural state, and training-related plasticity mechanisms, rather than producing broad and robust behavioral improvements outside a task context (Sathappan et al., 2019; Antal et al., 2022). At the same time, rigorously designed large-scale ACT studies have suggested that although cognitive training itself can lead to improvements, active stimulation does not consistently produce additional advantages on primary outcomes (Hausman et al., 2023; Horne et al., 2021).

The overall effect observed in the present study falls within the small-effect range, but its statistical significance and consistency across analytic settings remain meaningful. First, this suggests that the potential benefit of tDCS combined with cognitive training was not driven solely by a few extreme findings, but instead showed a relatively consistent positive direction across multiple studies. Second, in the field of cognitive training, particularly working memory training, transfer effects are typically weak and limited in stability. Therefore, a small positive pooled estimate in this context may still be informative (Nguyen et al., 2019; Pappa et al., 2020). A more appropriate interpretation is not to overstate this result as evidence for a universally applicable “enhancement” effect, but rather to view it as indicating that, under certain conditions, tDCS may provide a small additional benefit to training-related gains.

### 4.2 Boundaries of the effect: outcome type, time dimension, and training type

One of the most noteworthy patterns in the present study was that the pooled effect was statistically significant for trained-task outcomes, whereas untrained working-memory outcomes showed a smaller, non-significant positive effect. This pattern is consistent with the possibility that tDCS combined with cognitive training may more readily enhance performance gains that are closely aligned with the practiced task and its underlying cognitive processes. However, the direct contrast between trained and untrained outcomes did not reach statistical significance (*p* = 0.076), and the trained-task estimate was based on only nine studies. The findings therefore indicate a potentially stronger task-specific effect, rather than establishing a definitive difference between the two outcome categories. This pattern is broadly consistent with cognitive-training research showing that improvements are typically most evident on trained or closely related tasks, whereas transfer to other measures depends on the extent to which tasks share processing demands, strategies, and neural substrates (Nguyen et al., 2019; Pappa et al., 2020).

The interpretation of untrained outcomes also depends on the similarity between the training and assessment tasks. In the present review, an outcome was classified as untrained when the task had not been directly practiced during the intervention. This category therefore included both closely related paradigms and more distinct working-memory measures, rather than representing a uniform level of transfer distance. Accordingly, the present findings indicate that effects on unpracticed working-memory measures were smaller and less consistent than effects on trained tasks, but they do not provide a direct test of near versus far transfer. From a mechanistic perspective, if tDCS primarily facilitates task-related neural activity, learning efficiency, or post-training consolidation, its effects may be most apparent when the outcome measure engages processes that overlap closely with those activated during training. This interpretation is consistent with state-dependent accounts, which emphasize the interaction among task demands, current brain state, and individual baseline ability (Hsu et al., 2016; Sathappan et al., 2019). Repeated training paired with tDCS may also induce microstructural and functional plasticity, although such neural changes may not translate uniformly into behavioral improvements across dissimilar tasks (Antonenko et al., 2023).

With respect to the time dimension, the immediate post-test effect was statistically significant, while the follow-up estimate remained positive and was similar in magnitude, although it lay at the conventional alpha threshold. The lack of a significant difference between the two time points suggests that there was no clear evidence of a marked reduction in the effect at follow-up. At the same time, this result alone cannot confirm long-term maintenance, particularly given the smaller number of studies contributing follow-up data and the uncertainty surrounding the estimate. Previous large and rigorously designed trials have likewise shown that the long-term superiority of active over sham stimulation is not consistently observed, even when training-related gains are maintained (Hausman et al., 2023; Horne et al., 2021). Taken together, the current evidence is compatible with the possibility that a small additional benefit may extend beyond the immediate post-training period, while the duration and stability of this effect require further investigation using prospectively defined and standardized follow-up assessments.

The analysis of training type also warrants cautious interpretation. The effect reached statistical significance in the working memory training group, whereas the non-working-memory or mixed training group showed a positive effect of similar direction but did not reach significance; moreover, the difference between the two groups was almost zero. This suggests that the current evidence does not support training type as a significant moderator of the effect of active tDCS combined with cognitive training. In other words, the present results do not indicate that the additional effect of active tDCS is restricted to working memory training contexts, nor do they support the conclusion that working memory training reliably produces greater gains than other forms of cognitive training. A more plausible explanation is that the magnitude of the intervention effect may depend less on the categorical label of the training type itself and more on whether effective functional coupling is achieved among the training task, stimulation target, task load, and the individual’s current state (Hsu et al., 2016; Sathappan et al., 2019). This interpretation is also consistent with reviews of combined cognitive training and tDCS across clinical domains, which suggest that the critical issue is not the broad category of training per se, but whether the training content, stimulation target, and outcome measurement are clearly aligned (Burton et al., 2023).

### 4.3 Potential sources of heterogeneity: stimulation timing, age group, and individualized dosage

In the exploratory moderator analyses, the present study did not observe a significant moderating effect of stimulation timing. Although the effect reached statistical significance under online stimulation, the difference between online and non-online stimulation was not significant. This does not necessarily mean that stimulation timing is unimportant. Rather, it may indicate that existing studies differ substantially in how stimulation timing is defined, how tasks are arranged, and how long stimulation is delivered, making it difficult to identify stimulation timing as a stable and independent moderator. Previous studies have generally emphasized the state-dependent nature of tDCS, suggesting that the degree of alignment between stimulation and task-related processing may influence both the direction and magnitude of the effect. However, the online versus offline distinction itself has not shown a simple and consistent pattern (Hsu et al., 2016; Oldrati et al., 2018; Sathappan et al., 2019).

The analysis by age group also did not show a significant moderating effect. Although the effect was statistically significant in young adults but not in older adults, the point estimates were in the same direction and the between-group difference was not significant. This pattern is therefore more likely to reflect differences in the number of studies and statistical power than a genuine age-related dissociation. It should be noted that aging is often accompanied by brain atrophy, differences in conductive properties, changes in network state, and differences in baseline ability. As a result, the same nominal stimulation parameters may not produce equivalent intracranial electric fields across individuals (Opitz et al., 2015; Perceval et al., 2016; Meinzer et al., 2024). From this perspective, the absence of a significant age-group moderator may indicate that existing studies have not yet adequately controlled the critical pathway linking individualized dosage, intracranial electric fields, and behavioral responses.

This point may also help explain why positive trials, null findings, and failed replications have all appeared in recent years. A preregistered conceptual replication study showed that stimulation of either the posterior parietal cortex or the DLPFC failed to improve visual working memory capacity, thereby highlighting methodological issues and reproducibility challenges in tDCS cognition research (Jiang et al., 2024). In contrast, studies that focus on specific populations, specific stimulation targets, or longer intervention protocols may still observe localized positive effects. This suggests that the key question in the field is not simply whether tDCS “works,” but under what conditions it works, for whom it works, and through which processes it works.

### 4.4 Strength of evidence, limitations, and future directions

An important strength of the present study is that the core findings remained stable across multiple sensitivity analyses. This indicates that the overall results did not depend on a single assumed within-study correlation or a specific analytic pathway. Given that tDCS research has long been challenged by substantial effect heterogeneity, limited sample sizes, and a large parameter space, obtaining directionally consistent findings across different analytic settings strengthens the credibility of the present conclusions.

However, “robust” does not mean “definitive.” In recent years, methodological and reproducibility issues in tDCS research have been repeatedly discussed, including insufficient reporting of stimulation parameters, limited dose control, small sample sizes, the complexity of blinding and sham control, and potential interference from expectancy effects (Bikson et al., 2018; Woods et al., 2016; Meinzer et al., 2024). In particular, the sham condition and participants’ subjective judgments about stimulation condition may themselves become additional sources of variability (Fonteneau et al., 2019; Hooyman et al., 2024). Therefore, the conclusions of the present study are best understood as follows: the current evidence indicates a small positive pooled estimate, but its magnitude and boundary conditions remain uncertain. It should not be interpreted as a strong conclusion that has already been definitively established.

Several limitations should be acknowledged. First, the included studies varied in training tasks, stimulation parameters, intervention dose, and outcome measures, contributing to heterogeneity in the pooled estimates. Although RVE accounted for within-study dependency, the synthesis remained dependent on the quality of primary-study reporting, and several moderator analyses involved small and uneven numbers of studies. Second, only one of the 40 study-outcome entries was rated as low risk of bias, while most were rated as having some concerns. Although the overall estimate remained stable after excluding high-risk entries, methodological uncertainty remained. Third, the review was not prospectively registered, and no public protocol was archived. Although a second reviewer independently reassessed random subsets with high agreement, the full review process was not conducted entirely in duplicate. Fourth, the search was limited to PubMed, Scopus, Web of Science, and reference lists, so relevant unpublished or difficult-to-locate studies may have been missed. Finally, the trained-versus-untrained classification combined outcomes with different degrees of transfer, preventing clear conclusions about near versus far transfer.

Future research can advance this field in at least four directions. First, studies should more clearly distinguish trained-task outcomes, near-transfer outcomes, and far-transfer outcomes, and should systematically report results at each level rather than selectively reporting significant findings. Only when outcome levels are clearly defined can the generalization boundaries of tDCS combined with training be properly evaluated (Nguyen et al., 2019; Pappa et al., 2020). Second, future research should move beyond uniform nominal dosing and place greater emphasis on individualized intracranial electric fields. Existing evidence suggests that electric field strength, direction, and alignment with the target region may be closely related to behavioral gains and responder/non-responder differences. Therefore, MRI-based electric field modeling and individualized dosing may be key pathways for improving effect stability (Albizu et al., 2020; Caulfield et al., 2022; Padberg et al., 2021). Third, neuroimaging and other biomarkers should be integrated more systematically to identify which individuals are more likely to benefit from stimulation paired with training, which network processes are altered by stimulation, and how these neural changes mediate behavioral gains. Relying solely on behavioral endpoints is insufficient to explain the substantial variability in tDCS responses, whereas multimodal mechanistic evidence may improve interpretability (Antonenko et al., 2023; Meinzer et al., 2024). Fourth, the quality of trial design and reporting should be further improved, particularly with respect to sample size planning, procedural standardization, sham design, expectancy assessment, and preregistration. More convincing evidence in the future is likely to come from large-sample, multicenter, preregistered studies with long-term follow-up, rather than from the continued accumulation of small-sample, single-site trials with highly dispersed parameter settings (Bikson et al., 2018; Woods et al., 2018; Horne et al., 2021). From a longer-term perspective, the key progress in this field may not lie in repeatedly testing the simple contrast between active and sham stimulation, but in establishing a more refined matching framework among task characteristics, individual differences, stimulation parameters, and brain mechanisms.

## 5 Conclusion

Overall, active tDCS combined with cognitive training produced a small but statistically significant additional improvement in post-training working memory performance compared with sham tDCS combined with the same training. The effect was numerically larger for trained-task outcomes than for untrained working-memory outcomes, although the direct contrast between the two categories did not reach statistical significance and the trained-task estimate was based on a smaller number of studies. The follow-up estimate remained positive and was similar in magnitude to the immediate post-test effect, but was borderline at the conventional alpha threshold, making evidence for longer-term maintenance less conclusive. Stimulation timing, age group, and training type did not significantly moderate the overall effect. Taken together, these findings suggest that tDCS may provide a small adjunctive benefit when combined with cognitive training, with the clearest evidence currently observed for training-related gains. Further research is needed to clarify the extent of transfer to untrained tasks and the duration of benefit over time.

## Data Availability

The original contributions presented in the study are included in the article/Supplementary material, further inquiries can be directed to the corresponding authors.
