# Supplementary material for: Transcranial direct current stimulation combined with cognitive training for working memory in healthy adults: a systematic review and robust variance meta-analysis of trained and untrained outcomes
Source: Front Hum Neurosci. 2026 Aug 3;20:1902938. doi: 10.3389/fnhum.2026.1902938 (PMC13478250; doi:10.3389/fnhum.2026.1902938)
Supplement: Supplementary file 1 [file Data_Sheet_1.ZIP › Supplementary/Supplementary_File_1_Search_Strategy.docx]

**Search Strategy**

*Transcranial Direct Current Stimulation Combined With Cognitive Training for Working Memory in Healthy Adults: A Systematic Review and Robust Variance Meta-analysis*

**Overview**

This supplementary file provides the literature search strategy used for the systematic review and meta-analysis. Systematic searches were conducted in PubMed, Scopus, and Web of Science from database inception to January 2026. A broad-search and refined-screening strategy was adopted to reduce the risk of missing studies that reported working-memory outcomes without explicitly using the term “working memory” in the title, abstract, or keywords. Therefore, working-memory-related search terms were not included in the initial database search; eligibility for working-memory outcomes was determined during screening and data extraction.

**Table S1. Database search strategy**

| **Database** | **Search coverage** | **Search date / time span** | **Search string** | **Records retrieved** |
| --- | --- | --- | --- | --- |
| PubMed | Database inception to January 2026 | January 2026 | ("transcranial direct current stimulation" OR "tDCS") AND ("practice" OR "training" OR "cognitive intervention") | 1,787 |
| Scopus | Database inception to January 2026 | January 2026 | TITLE-ABS-KEY("transcranial direct current stimulation" OR "tDCS") AND TITLE-ABS-KEY("practice" OR "training" OR "cognitive intervention") | 2,859 |
| Web of Science | Database inception to January 2026 | January 2026 | TS=("transcranial direct current stimulation" OR "tDCS") AND TS=("practice" OR "training" OR "cognitive intervention") | 2,592 |

**Note.** The PubMed search was conducted using the All Fields search setting. Scopus and Web of Science syntax was adapted using standard field tags.

**Supplementary search procedures**

The reference lists of all included studies and relevant reviews were screened manually to identify additional potentially eligible reports. No automation tools were used to exclude records before screening. All records retrieved from database searches were imported into reference management software, and duplicates were removed before title/abstract screening.

**Table S2. Search and screening summary**

| **Item** | **Number** |
| --- | --- |
| Records identified from PubMed | 1,787 |
| Records identified from Scopus | 2,859 |
| Records identified from Web of Science | 2,592 |
| Total records identified from databases | 7,238 |
| Duplicate records removed | 3,521 |
| Records removed for other reasons before screening | 0 |
| Records screened | 3,717 |
| Records excluded during title/abstract screening | 3,659 |
| Reports sought for retrieval | 75 |
| Reports not retrieved | 3 |
| Reports assessed for eligibility | 72 |
| Studies included in review and meta-analysis | 23 |

**Note.** The counts are reported to be consistent with the PRISMA flow diagram.

**Table S3. Reasons for exclusion at full-text screening**

| **Reason for exclusion** | **Number of reports** |
| --- | --- |
| Reviews | 5 |
| No usable data or no behavioral outcomes | 21 |
| Ineligible grouping or control condition | 5 |
| Duplicate reports | 4 |
| No cognitive training | 3 |
| Additional interventions included | 4 |
| tDCS not used experimentally | 2 |
| Methodological papers or protocols | 4 |
| Cognitive impairment as intervention aim | 1 |

**PRISMA flow diagram**

The full study identification, screening, eligibility assessment, and inclusion process is shown in Figure 1 of the main manuscript.
