# Supplementary material for: Transcranial direct current stimulation combined with cognitive training for working memory in healthy adults: a systematic review and robust variance meta-analysis of trained and untrained outcomes
Source: Front Hum Neurosci. 2026 Aug 3;20:1902938. doi: 10.3389/fnhum.2026.1902938 (PMC13478250; doi:10.3389/fnhum.2026.1902938)
Supplement: Supplementary file 1 [file Data_Sheet_1.ZIP › Supplementary/Supplementary_File_3_R_Code/results/Figure_3_forest_plot_from_R.pdf]

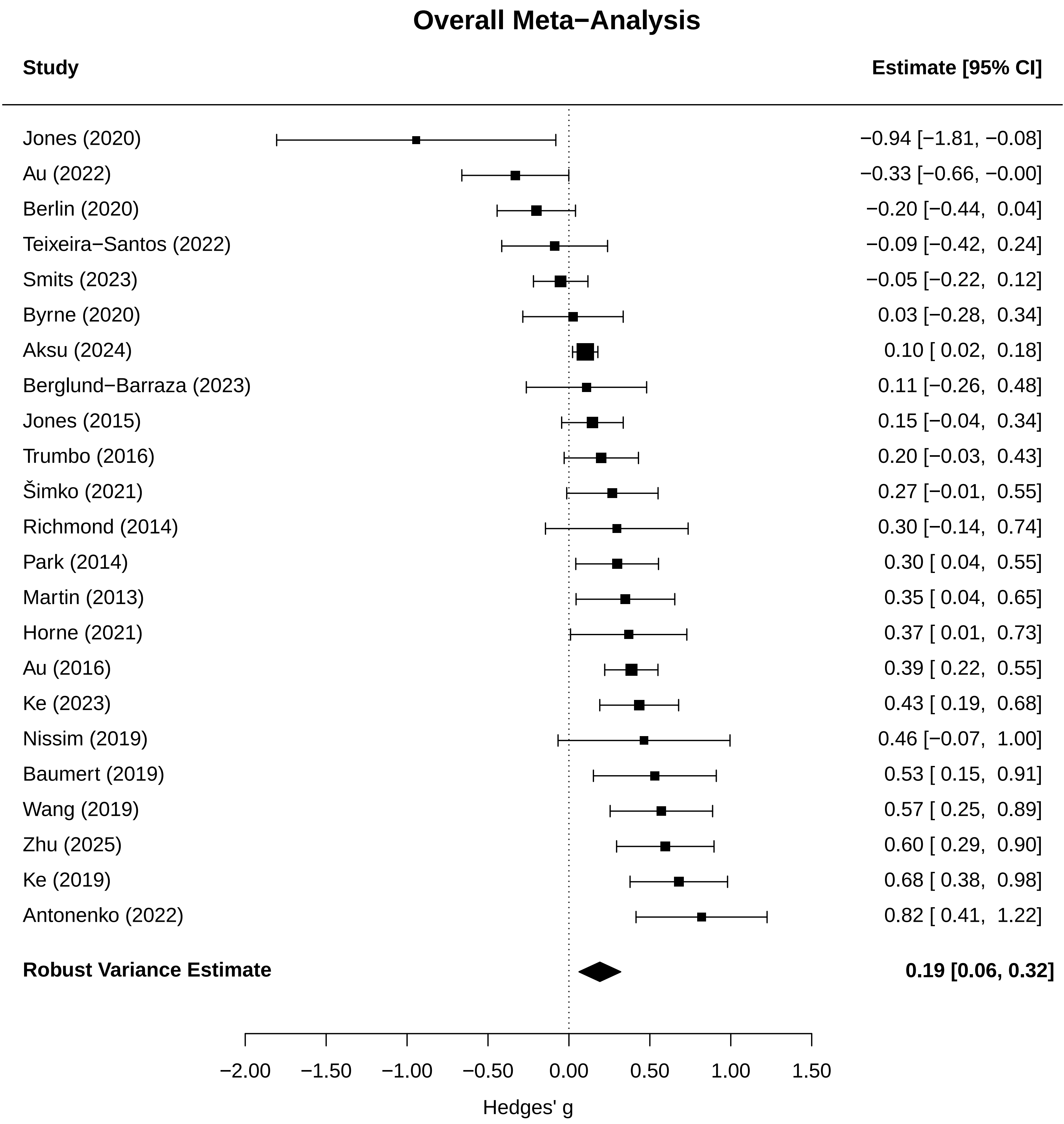
Overall Meta−Analysis
Study
Estimate [95% CI]
Jones (2020) −0.94 [−1.81, −0.08]
Au (2022) −0.33 [−0.66, −0.00]
Berlin (2020) −0.20 [−0.44, 0.04]
Teixeira−Santos (2022) −0.09 [−0.42, 0.24]
Smits (2023) −0.05 [−0.22, 0.12]
Byrne (2020) 0.03 [−0.28, 0.34]
Aksu (2024) 0.10 [0.02, 0.18]
Berglund−Barraza (2023) 0.11 [−0.26, 0.48]
Jones (2015) 0.15 [−0.04, 0.34]
Trumbo (2016) 0.20 [−0.03, 0.43]
Šimko (2021) 0.27 [−0.01, 0.55]
Richmond (2014) 0.30 [−0.14, 0.74]
Park (2014) 0.30 [0.04, 0.55]
Martin (2013) 0.35 [0.04, 0.65]
Horne (2021) 0.37 [0.01, 0.73]
Au (2016) 0.39 [0.22, 0.55]
Ke (2023) 0.43 [0.19, 0.68]
Nissim (2019) 0.46 [−0.07, 1.00]
Baumert (2019) 0.53 [0.15, 0.91]
Wang (2019) 0.57 [0.25, 0.89]
Zhu (2025) 0.60 [0.29, 0.90]
Ke (2019) 0.68 [0.38, 0.98]
Antonenko (2022) 0.82 [0.41, 1.22]
Robust Variance Estimate 0.19 [0.06, 0.32]
−2.00
−1.50
−1.00
−0.50
0.00
0.50
1.00
1.50
Hedges' g
